# Performance evaluation of point-of-care test for detection of *Cryptosporidium* stool antigen in children and HIV infected adults

**DOI:** 10.1186/1756-3305-7-227

**Published:** 2014-05-16

**Authors:** Techalew Shimelis, Endale Tadesse

**Affiliations:** 1 Department of Medical Laboratory Science, College of Medicine and Health Sciences, Hawassa University, Hawassa, Ethiopia

**Keywords:** Evaluation, RDT, *Cryptosporidium*

## Abstract

**Background:**

Gastro-enteritis is associated with significant morbidity and mortality in patients with HIV/AIDS and children, and *Cryptosporidium* is the most important parasite implicated. To date, several commercial companies have developed simple and rapid point-of-care tests for the detection of *Cryptosporidium* infection; however, information is scarce regarding their diagnostic significance in Ethiopia. This study aimed at evaluating the performance of a rapid diagnostic test (RDT) for the detection of *Cryptosporidium* stool antigen.

**Methods:**

A hospital-based cross-sectional study was conducted in Hawassa University Hospital, southern Ethiopia from May to November 2013. Faecal samples were collected from a total of 100 children and 250 HIV infected individuals with diarrhea or CD4 T-cell count lower than 200 cells/μl. Specimens were processed using direct, formol-ether concentration and modified Ziehl-Neelsen techniques for diagnosis of *Cryptosporidium* and other parasites. One hundred faecal samples (50 positives for *Cryptosporidium*, 35 positives for other parasites and 15 negatives for any intestinal parasites) were tested using the CoproStrip™*Cryptosporidium* kit (Savyon Diagnostics Ltd, Israel). Test parameters were calculated using microscopy of the modified Ziehl-Neelsen stained stool smear as reference method.

**Results:**

The performance of the RDT was first compared to routine microscopic analysis (examination ≤10 min). The CoproStrip™*Cryptosporidium* RDT correctly detected 31 of 42 positive samples and 49 of 50 negative samples (i.e., 11 false negatives and 1 false positive). Sensitivity, specificity, PPV, NPV and accuracy were calculated to be 74, 98, 97, 84 and 88%, respectively. Upon thorough microscopic analysis (examination >10 min), 8 more samples with very low oocyst density were found. However, these were missed by the kit and lower the sensitivity and NPV to 62 and 72%, respectively. No cross-reactivity was observed with any of the helminthic or other protozoan parasites including *Isospora* and *Cyclospora* species.

**Conclusion:**

Based on the results described herein, the CoproStrip™*Cryptosporidium* test could be used as an alternative to conventional microscopy especially where diagnosis of *Cryptosporidium* is limited due to time constraints, lack of experienced microscopists or unavailability of appropriate equipment/electricity.

## Background

*Cryptosporidium* is a ubiquitous protozoan parasite that infects humans and a wide range of domestic and wild animals [1]. Transmission of *Cryptosporidium* is mainly through fecal-oral route, as well as through contaminated water and food, person-to-person spread and contact with infected animals [2,3]. In developing countries where there is low hygiene level, poor sanitation, no good water management, and frequent contact with animals, the burden of cryptosporidiosis remains to be a major health problem [4]. In Ethiopia, *Cryptosporidium* prevalence was shown to range from 3.1% to 25.9% in HIV/AIDS patients [5-9] and 3.3% -12.2% in children [10-12]. The weaker immune function in HIV/AIDS patients and children attributes for higher prevalence and clinical impact in these groups. In immunodeficient patients, intestinal cryptosporidiosis is characterized by severe and chronic diarrhea, which leads to dehydration, wasting and often death [13,14]. Similarly, *Cryptosporidium* associated gastroenteritis in early childhood may cause malnutrition, impaired physical and cognitive development, and death [4]. No effective therapy is available to treat *Cryptosporidium* infection, but antiretroviral therapy (ART) restores immunity and reduces cryptosporidiosis-related morbidity in HIV/AIDS patients [15].

Timely and accurate diagnosis of *Cryptosporidium* is important to properly manage infected individuals and understand its epidemiology for effective prevention. Microscopic examination of the modified Ziehl-Neelsen stained stool smear is a conventional method for diagnosis of *Cryptosporidium*. This method has excellent specificity (98-100%) though its sensitivity (75-84%) is inferior to techniques such as direct fluorescent-antibody tests (DFA), polymerase chain reaction (PCR), and enzyme linked immunosorbent assay (ELISA) [16-18]. Unlike the ELISA and PCR techniques, which may not distinguish between active and resolved infections, microscopy has the advantage of indicating active infections [17].

Although *Cryptosporidium* is endemic in Ethiopia, its diagnosis is limited mainly due to the unsuitability of the methods in most of our contexts. In the recent years, several commercial companies have developed rapid diagnostic tests (RDTs) that are simple to perform, applicable in various settings and have short test time compared to the conventional microscopy for detecting *Cryptosporidium*. However, as these products demonstrate varying performances, they require extensive evaluations in diverse field conditions in order to evaluate their diagnostic usefulness. Considering its market availability and easier applicability, we determined the performance characteristics of the RDT, CoproStrip™*Cryptosporidium* (Savyon Diagnostics Ltd, Israel) against modified Ziehl-Neelsen microscopy so as to realize whether this RDT offers alternatives to conventional diagnosis.

## Methods

### Participants and samples

This hospital-based cross-sectional study was conducted using stool samples collected from a consecutive 100 children with diarrhea and 250 HIV infected individuals with diarrhea or CD4 T-cell count lower than 200 cells/μl at Hawassa University Hospital, southern Ethiopia from May to November 2013. In total, 100 stool samples were considered for analysis; 50 of which were positive for *Cryptosporidium* oocyst. Out of 50 samples negative for *Cryptosporidium* oocyst, 35 had various parasites: *Giardia lamblia* (n = 16), *Entamoeba histolytica/dispar* (n = 8), *Ascaris lumbricoides* (n = 5), *Isospora belli* (n = 5), *Strongyloides stercoralis* (n = 4), *Cyclospora* species, *Blastocystis hominis*, *Endolimax nana*, *Entamoeba coli*, *Trichuris trichiura*, *Taenia* species*, Schistosoma mansoni*, *Hymanolopis* species and hookworm (n = 2 each). In the remaining 15 samples, no parasite was detected. Participants treated for any intestinal parasites during the month prior to the study were excluded. The study obtained ethical clearance from the Institutional Review Board of Hawassa University College of Medicine and Health Sciences. Participation was fully voluntary and informed consent/assent was obtained from each study participant/parents/guardians. Physicians managed those participants found to be infected with pathogenic parasites.

### Microscopy

About 2 grams of single stool sample was collected from each participant using clean, dry and screw-capped cups. Fresh samples were processed using direct (saline and iodine mounts) and formol-ether concentration techniques for non-coccidian intestinal parasites. The sediment of each concentrated stool samples were processed using the modified Ziehl-Neelsen technique for microscopic examination of *Cryptosporidium*[19]. In this technique, air-dried stool smears were fixed with methanol for 3 minutes, stained by carbol fuchsine for 15 minutes, decolorized with 1% acid alcohol for 15 seconds, and counter stained with 0.5% methylene blue for 30 seconds. Stained smears were air-dried and examined microscopically (using 100x objective) for oocysts of *Cryptosporidium*. We assumed that microscopists scan slides routinely for about 10 minutes before they declare negative results [20]. Thus, we considered results within this reading time to analyze the RDT’s performance. However, the entire smear for all negative slides was scanned for an extended time (>10 minutes) in order not to miss any positive sample.

### Rapid diagnostic test

Fresh stool samples were tested for *Cryptosporidium* antigen using the CoproStrip™*Cryptosporidium* (Savyon Diagnostics Ltd, Israel) RDT kit as specified by the manufacturer. The CoproStrip™*Cryptosporidium* is a rapid chromatographic immunoassay for the qualitative detection of *Cryptosporidium* antigens in human faeces. The test procedure used involved emulsification of a stool sample the size of a small pea (150 mg or 150 μl) in 1 ml of buffer; 4 drops of the mixture were dispensed into the specimen well. The results were read at 10 minutes. A positive test result is indicated when control (green colored) and test (red colored) lines are visible (regardless of color intensity), and a negative result is when only the control line is visible. Absence of the control line indicates invalid results and testing were repeated in those instances. An expert microscopist read all slides and a laboratory technologist blinded to the microscopy results performed the RDT. The principal investigator resolved any discordant results, and further checked all positive slides and 10% of the negative slides. Data entry and analysis was performed using STATA Version-10, and a descriptive summary was presented. Test parameters for the RDT including sensitivity, specificity, positive predictive value (PPV), negative predictive value (NPV), and accuracy were determined using the modified Ziehl-Neelsen microscopy as reference method. Cohen’s kappa was calculated to show the degree of agreement between the RDT and the modified Ziehl-Neelsen microscopy.

## Results

A total of 350 stool specimens were investigated for different parasites using microscopy; of which, 250 were from HIV infected individuals and 100 were from children. Oocysts of *Cryptosporidium* were detected in 42 samples (32 HIV patients and 10 children) within 10 minutes reading time. Upon thorough microscopic analysis (examination >10 min), 8 more samples from HIV infected participants were found to have rare parasites (1–4 oocysts). Various helminthic and protozoan parasites were also detected in most stool samples.

The diagnostic performance characteristics of the CoproStrip™*Cryptosporidium* test kit against the modified Ziehl-Neelsen microcopy is summarized in Table 1. The RDT correctly detected 31 of the 42 samples positive by microscopy. Eleven samples were false negative even though oocysts were not uncommon in the modified Ziehl-Neelsen stained smear. Moreover, results from 49 of 50 microscopy negative specimens were concordant, yielding an overall RDT’s accuracy of 88%. The sensitivity, specificity, PPV, and NPV were calculated to be 74, 98, 97 and 84%, respectively. However, the kit failed to detect *Cryptosporidium* antigen in those 8 stool samples with rare oocysts, and the sensitivity and NPV lowered to 62 and 72%, respectively. Analysis using kappa statistic showed the presence of diagnostic agreement between the modified Ziehl-Neelsen microcopy and the RDT, in which the degree of agreement was higher for ≤10 min reading time (Kappa = 0.75; 95% CI: 0.61- 0.88) compared to >10 min reading time (Kappa = 0.60; 95% CI: 0.45- 0.75). The RDT did not show cross-reactivity with any of the other tested parasites including *Isospora belli* and *Cyclospora* species.

**Table 1:** **The diagnostic performance characteristics of the CoproStrip™** ***Cryptosporidium *** **against the modified Ziehl-Neelsen microscopy, southern Ethiopia, 2013**

**Modified Ziehl-Neelsen microscopy**	**No of samples**	**No positives by RDT**	**Sensitivity (%)**	**Specificity (%)**	**PPV (%)**	**NPV (%)**
Positive						
Examination ≤10 min	42	31	74	98	97	84
Examination >10 min	50	31	62	98	97	72
Negative	50	1				

RDT, rapid diagnostic test; PPV, Positive Predictive Value; NPV, Negative Predictive Value.

## Discussion

There is a greater demand for efficient diagnostic methods for *Cryptosporidium* in developing nations where the infection is prevalent and most consequential. In an attempt to find alternative testing methods to traditional microscopy, which has limited diagnostic suitability in resource-limited countries, we evaluated the commercial product, CoproStrip™*Cryptosporidium*. This point-of-care test was designed to detect *Cryptosporidium* antigen from stool specimens, and we determined its diagnostic performance characteristics against microscopy of the modified Ziehl-Neelsen stained smear. The sensitivity, specificity, PPV, and NPV of the test kit were 74, 98, 97 and 84%, respectively.

Studies have shown varying performance levels of RDTs, which might be due to differences in commercial products, dissimilar methodologies employed, and genetic diversity of *Cryptosporidium* with geographical regions. Though the manufacturer for CoproStrip™*Cryptosporidium* kit claimed higher sensitivity (>99%), our observation was comparable with that reported for RIDAQuick *Cryptosporidium* (R-biopharm Diagnostic) (62-72%) [21,22], Remel-Xpect *Cryptosporidium* (Remel Inc.) (69%) [22], and ImmunoCard STAT! *Cryptosporidium/Giardia* (Meridian Bioscience Inc.) (68-71%) [16,22]. A contrasting lower (47.2%) [22] and higher (98%) [23] sensitivity was also reported for the product, Crypto-Strip (Coris BioConcept). As to the specificity, findings seem to be consistent that RDTs have excellent performance in detecting samples with no *Cryptosporidium* parasite [16,21-23]. Similarly, the current study showed excellent specificity for CoproStrip™*Cryptosporidium* kit in a context where various intestinal parasites are endemic, as also claimed by the manufacturer.

It was noted that some samples tested negative with the kit had abundant *Cryptosporidium* oocysts. As to whether *Cryptosporidium* species/subtypes in our study area contributed to the observed false negative results was unclear as the organisms were not further characterized. However, previous studies showed the lower sensitivity of RDTs in detecting non- *C. parvum/C. hominis*[22,23] as well as the slightly better performance for *C. hominis* compared to *C. parvum*[22] infections. Moreover, the sensitivity of the CoproStrip™*Cryptosporidium* kit seems to be influenced by parasite density where false negative results more likely occurred in samples with fewer numbers of oocysts as also reported by others [16,22]. This may also be attributed to the simultaneous lower antigen concentration in those samples. The excellent positive predicative value shown for this test kit indicates the reliability of positive results to truly detect those with *Cryptosporidium* infection. Nevertheless, the observed false positive result in one patient may be due to the persistent antigen shading in recently cured cases or in a situation of intermittent oocyst excretion.

Microscopy of the modified Ziehl-Neelsen stained smear is useful in diagnosing current *Cryptosporidium* infection and has the added advantage of detecting other parasites such as *Cyclospora cayetanensis* and *Isospora belli*. However, microscopy is technically demanding and has applicability challenges to be established in resource-constrained settings. The CoproStrip™*Cryptosporidium* kit, which is simple to test, has short test time (10 min) and does not require laboratory equipment or electricity, addresses the inherent challenges of microscopy. In this regard, the kit improves the efficiency of laboratories by reducing labor, time and resources; thus, has great significance in screening large populations such as HIV infected people, children, and in outbreak scenarios. It should be noted that an RDT with such performance is not to replace microscopy, but rather to serve as a diagnostic option in situations where microscopy is not suitable. The need to confirm negative RDT test results with more sensitive tests in patients that remain symptomatic should also be remembered.

The limitations for this study include the tests employed for diagnosis of *Cryptosporidium* did not include more accurate methods such as PCR and DFA, against these comparisons of the RDT test is imperative. Moreover, failure to characterize the *Cryptosporidium* parasite using molecular techniques in those false negative samples might be a missed opportunity to better elucidate the findings in a way to offer inputs towards continuous improvement of RDT’s performance.

## Conclusion

The CoproStrip™*Cryptosporidium* test could be used as an alternative to conventional microscopy especially where diagnosis of *Cryptosporidium* is limited due to time constraints, lack of experienced microscopists or unavailability of appropriate equipment/electricity. In the Ethiopian context, where diagnostic options are limited, this point-of-care test could be imported and utilized for screening *Cryptosporidium* in high risk individuals such as HIV patients and children so that its clinical impact could be minimized. Investigating RDTs performance in relation to the molecular diversity of *Cryptosporidium* in a given geographical region might be important to further improve the performance of test products.

## Competing interests

The authors declare that they have no competing interests.

## Authors’ contributions

TS designed and carried out the laboratory work; TS and ET performed the statistical analyses, interpretation and contributed to the write up. Both authors read and approved the final version of the manuscript.
